# Experimental Investigation of Changes in Performance, Emissions, and Engine Vibration in Diesel Engines Using Four Fuel Combinations of Diesel, Biodiesel, Octanol, and Hydrogen

**DOI:** 10.1021/acsomega.6c04041

**Published:** 2026-09-10

**Authors:** Volkan Sabri Kül, Mehmet Parlak, Mehmet Sarıtaş, Selahaddin Orhan Akansu

**Affiliations:** † Graduate School of Natural and Applied Sciences, (Department of Mechanical Engineering), 52958 Erciyes University, Kayseri 38280, Turkey; ‡ Department of Mechanical Engineering, Erciyes University, Kayseri 38280, Turkey; § Graduate School of Natural and Applied Sciences, (Department of Mechanical Engineering), Erciyes University, Kayseri 38280, Turkey

## Abstract

Energy efficiency is a vital concept in our modern world. The most efficient use of energy resources is crucial for sustainability. A significant portion of global energy consumption is attributed to the transportation sector. The efficient operation of internal combustion engines, which power the transportation sector, and the efficiency of the fuels they use are becoming increasingly important. Therefore, researchers continue their work to improve fuel efficiency and find alternative fuels. In this study, test fuels created from four-fuel combinations of diesel, biodiesel (obtained from a mixture of 10% spent coffee grounds oil and 90% waste cooking oil), octanol, and hydrogen were investigated at 150 N m (half load) and 300 N m (full load) to improve engine performance and emissions. The effect of the test fuels on engine vibrations was also examined using accelerometer sensors positioned on the horizontal and vertical axes of the diesel engine. Since this four-fuel combination and the investigation of its effects on engine vibration are very rare in the literature, this study fills an important gap. The main objective of this study was to investigate the impact of octanol and biodiesel blends with hydrogen reinforcement on engine performance, vibrations, and emissions. The study results showed that the highest brake thermal efficiency values were obtained with the D90O10 mixture at both low and high loads. The D90O10 test fuel achieved thermal efficiency of 27.45% at 150 N m load and 33.72% at 300 N m load. These values are higher than those of D100 (BTE; 26.43% at 150 N m load and 33.24% at 300 N m load). The most important finding of this study is that the addition of 10% octanol to pure diesel fuel increases thermal efficiency. Among the important findings regarding vibration, it is the fact that blended fuels containing biodiesel reduce vibration at low loads (150 N m) compared to pure diesel fuel. While the combined vibration of D100 at 150 N m is 51.861 mm/s^2^, it was determined that the mixtures containing octanol and biodiesel (D90O10 and D70B20O10) are below this value (at levels of 46.178 mm/s^2^ and 46.353 mm/s^2^, respectively). However, at high loads (300 N m), vibration increased in fuels containing biodiesel. Hydrogen supplementation was found to have an enhancing effect on vibration at both 150 N m and 300 N m loads. Biodiesel-containing test fuels generally improved carbon monoxide emissions.

## Graphical abstract

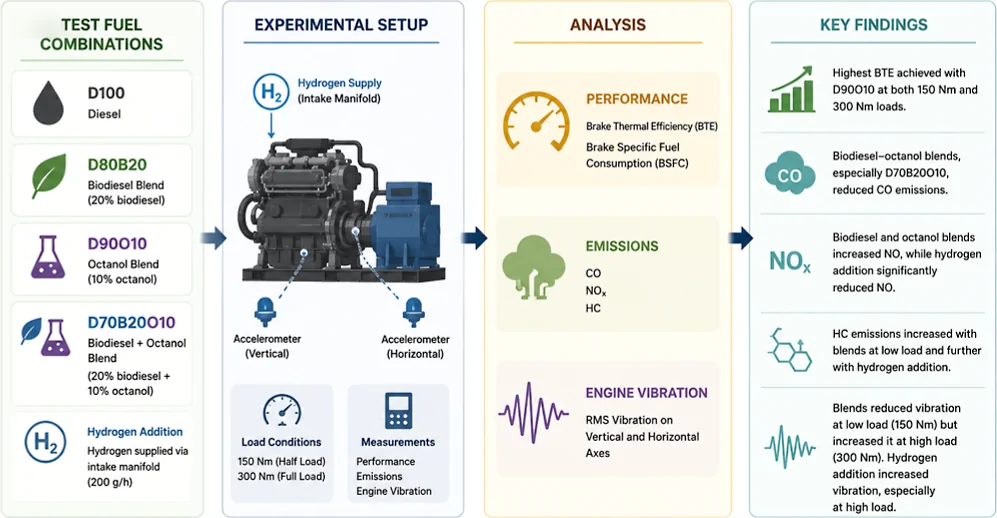

## 1 Introduction

Energy and efficiency are two crucial concepts shaping the modern world. In the modern world, these two concepts are even more prominent. In fact, the importance of these two concepts dates back to humankind’s discovery of fire and the invention of the wheel. For example, tree trunks (logs or stumps) used to move heavy loads around 3500 BC were developed over the years to meet various needs, eventually becoming the wheel, an indispensable component of modern vehicles. This historical example stands before us as the most striking illustration of how efficiency can lead to more beneficial use of energy. In fact, development is similar in today’s world. Power plants are constantly making new improvements, aiming to produce more energy with fewer energy resources. Similarly, energy-consuming vehicles also strive to produce more benefits with fewer resources. Energy and efficiency, as two concepts that mutually reinforce each other, will continue to increase in importance in the future. Undoubtedly, one of the most important machines that has changed our world through the efficient use of energy is the internal combustion engine. Diesel engines, in particular, are more efficient than spark-ignition engines and are therefore more prominent in transportation. Rudolf Diesel realized that the steam engine, the dominant heat engine of the time, had a very low efficiency of only 3% and wasted a large portion of fuel energy, compared to the ideal energy conversion cycle formulated by Carnot in 1824. As a result of his work on creating an efficient heat engine, Rudolf Diesel filed a patent application for a new and rational heat engine with the Imperial Patent Office in Berlin on February 27, 1892, thus securing the first diesel engine in history. Since that date, internal combustion engines have been gradually improved and have replaced the modern engines we see today.^1,2^ In this historical process, not only have engines improved in terms of their physical properties, but fuels have also improved in terms of thermal efficiency. It is clear that the simultaneous development of both engines and fuels has led to the modern fuels and engines we have today. The developments discussed above in broad terms have been achieved through the work of researchers. Therefore, research occupies a vital position in the development of our current equipment. To make internal combustion engines more efficient and reduce emissions, researchers are currently making significant contributions to the literature by developing alternative fuels. In this context, the efforts of researchers to develop alternative fuels by adding additives to fuels used in internal combustion engines are a crucial building block in shaping our future. For this purpose, experiments were conducted in this study to explore alternative fuels in a diesel engine. The experiments were carried out using various fuel combinations created from mixtures of diesel, biodiesel (biodiesel produced from spent coffee grounds oil and waste cooking oil mixtures), octanol, and hydrogen (H_2_) fuels. When the diesel engine was run with the various test fuels created, engine vibration data in the horizontal and vertical axes were measured to investigate how engine vibration changed with different fuel mixtures. The investigation of a four-fuel mixture of diesel, octanol, biodiesel, and hydrogen fuels and how this mixture causes changes in engine vibration is the originality of this study. Thus, it is believed that it will make a significant contribution to the literature.

Since studies investigating the relationship between four-component fuel mixtures and changes in engine vibration are limited, it is believed that this study will fill an important gap in the literature. Similar studies in the literature in recent years have been presented below.

Nour et al.^3^ investigated the changes in diesel engine performance by mixing alcohols such as butanol, heptanol, and octanol with diesel fuel. In this study, a single-cylinder diesel engine was used. The test engine was equipped with various sensors such as an in-cylinder pressure sensor, fuel flow meter, and temperature sensor to collect performance data. Test fuels were prepared by adding alcohol to the diesel fuel at volumetric percentages of 10% and 20%. Experiments were carried out at engine speeds of 900 and 1500 rpm and at loads of 0%, 25%, 50%, 75%, and 100%. As a result of the experiments, the butanol additive increased the thermal efficiency (Brake Thermal Efficiency (BTE)) compared to other test fuels. The thermal efficiency values of the octanol and heptanol mixed fuels were similar to the thermal efficiency of pure diesel fuel under most conditions. They reported a decrease in NO emissions and an increase in CO and HC emissions in all tested alcohol diesel mixtures. Nour et al.^5^ investigated the effect of pentanol/aqueous ethanol/diesel and octanol/aqueous ethanol/diesel ternary mixtures on the combustion characteristics of a compression ignition engine in another study. As a result of the experiments, they reported that the peak cylinder pressures for pentanol/ethanol/diesel (Pe10E10D80) and octanol/ethanol/diesel (Oc10E10D80) were lower than D100. They observed that the ignition delay and combustion duration increased for the ternary mixtures. According to the results of their study, the BTE value of Oc10E10D80 is higher than D100. In contrast, the BTE value of Pe10E10D80 is lower. They stated that the addition of pentanol and octanol to the aqueous ethanol/diesel mixture generally improved emissions. Ashok et al.^4^ investigated the effects of Calophyllum Inophyllum biodiesel and *n*-octanol on diesel engine performance. They prepared five different fuel mixtures with *n*-octanol concentrations ranging from 10% to 50% by volume. Increasing the *n*-octanol ratio in the mixtures resulted in higher peaks in in-cylinder pressure and heat release rates. It also caused an increase in ignition delay. Brake thermal efficiency improved with an increase in *n*-octanol ratio up to 30%, then began to decrease with further increases in *n*-octanol ratio in the mixtures. In addition, the cooling effect produced by a lower *n*-octanol ratio in the mixture lowered the in-cylinder temperature, resulting in lower NO emissions. However, the poor ignition and evaporation properties of *n*-octanol led to an increase in CO and smoke emissions. Çakmak et al.^6^ created fuel mixtures using diesel, biodiesel, and *n*-octanol at a constant speed of 1500 rpm and load values of 25%, 50%, 75%, and 100%. A diesel engine was operated with these fuel mixtures to investigate changes in engine performance and emissions. According to the experimental results, they observed that the highest thermal efficiency at 100% load was obtained with a three-fuel mixture prepared with 75% diesel, 20% biodiesel, and 5% octanol. However, a gradual decrease in thermal efficiency occurred with increasing octanol concentration in the mixture. Kumar et al.^7^ investigated the effects of mixing diesel with 30% by volume of iso-butanol, *n*-pentanol, *n*-hexanol, and *n*-octanol on the combustion and emission characteristics of a diesel engine. They reported improvements in CO, HC, and NO emissions in the octanol-mixed fuel. Ahn et al.^8^ investigated the applicability of *n*-octanol as an alternative fuel in diesel engines. To analyze the effects of *n*-octanol on engine performance and emissions, they conducted experiments using pure diesel and different *n*-octanol mixtures. According to the results obtained, they reported that diesel/n-octanol mixtures consistently improved thermal efficiency, while *n*-octanol increased specific fuel consumption due to its lower energy content. All *n*-octanol mixtures not only reduced NO emissions compared to pure diesel but also significantly improved CO, HC, and smoke opacity. They reported that *n*-octanol significantly increased engine efficiency but emphasized the importance of optimizing the *n*-octanol mixture ratio for performance improvements. Yousufuddin et al.^9^ used biodiesel and hydrogen fuels as additive fuels to conduct experiments in a diesel engine. As a result of their studies, they observed that injecting hydrogen into the combustion chamber along with an 80% diesel 20% biodiesel mixture increased thermal efficiency. With the addition of hydrogen, there was a decrease in HC emissions, while an increase in NO emissions was observed. Serin et al.^10^ used diesel, biodiesel, and hydrogen fuels in their study. According to their study, they observed that hydrogen improved fuel consumption. While a decrease in CO emissions was observed, an increase in NO emissions was observed. Kül et al.^11^ conducted experiments with fuel mixtures obtained from diesel, biodiesel, nanoboron, and hydrogen fuels. As a result of their experiments, they reported that the biodiesel additive had a negative effect on thermal efficiency, while the hydrogen additive showed a relative improvement. They observed an improvement in NO emissions in experiments using hydrogen.

In this study, alternative fuel blends consisting of diesel, octanol, biodiesel, and hydrogen were systematically developed with the aim of comprehensively improving compression ignition engine performance and exhaust emissions. Furthermore, the simultaneous effects of these four-fuel multiformulations on engine structural vibrations under varying engine loads were investigated in depth. While recent extensive literature studies, including significant studies such as refs 30–33 and refs 35–37 , have comprehensively elucidated the individual or binary behaviors of alternative fuel metrics, a critical research gap remains regarding the simultaneous evaluation of octanol, biodiesel, and hydrogen supplementation. This study explicitly addresses the fundamental academic problem of overcoming the complex balancing issues between optimizing Brake Thermal Efficiency (BTE) and reducing engine vibrations typically caused by alternative blends. Consequently, the key novelty and pioneering nature of this study is that it presents the first systematic investigation of how these delicate multifuel interactions act as a dual balancing element for both performance and structural engine dynamics. Thus, it fills a significant gap in the literature and provides unique, applicable information to the field of clean combustion systems.

## 2 Methodology

In this study, experiments were conducted to improve the performance and emissions of compression ignition engines by adding biodiesel (10% produced from SCGO and 90% WCO), octanol, and hydrogen to the diesel fuel. To analyze the results, engine power, engine speed, torque, exhaust temperatures, fuel flow rate, and both vertical and horizontal vibration measurements were collected. These data were analyzed, and the test variations were discussed.

Experiments were conducted at 50% (150 N m) and 100 N m (300 N m) engine loads and a constant engine speed of 660 rpm (the torque levels of 150 N m and 300 N m were selected to simulate typical partial-load and high-load engine operations, respectively. This selection enables a robust comparison of the impact of hydrogen enrichment across different operating regimes.) Pure diesel fuel was used as the first experimental setup, and other blended fuels were compared to pure diesel. Before each experimental setup, the test engine was adjusted to the test conditions and run for at least 15 min to allow the engine to stabilize. After the engine reached a stable state, experimental data collection began. The collected data were recorded by a performance and emission measurement system for analysis.

The subheadings of this section detail the experimental setup, the technical specifications of the equipment used for data collection, the properties of the fuels, and the processes for preparing the blended fuels.

### 2.1 Experimental Setup

Figure 1 shows a representative image of the experimental setup. The specifications and technical details of the equipment in the experimental setup are presented in detail in this section.

**Figure 1:**
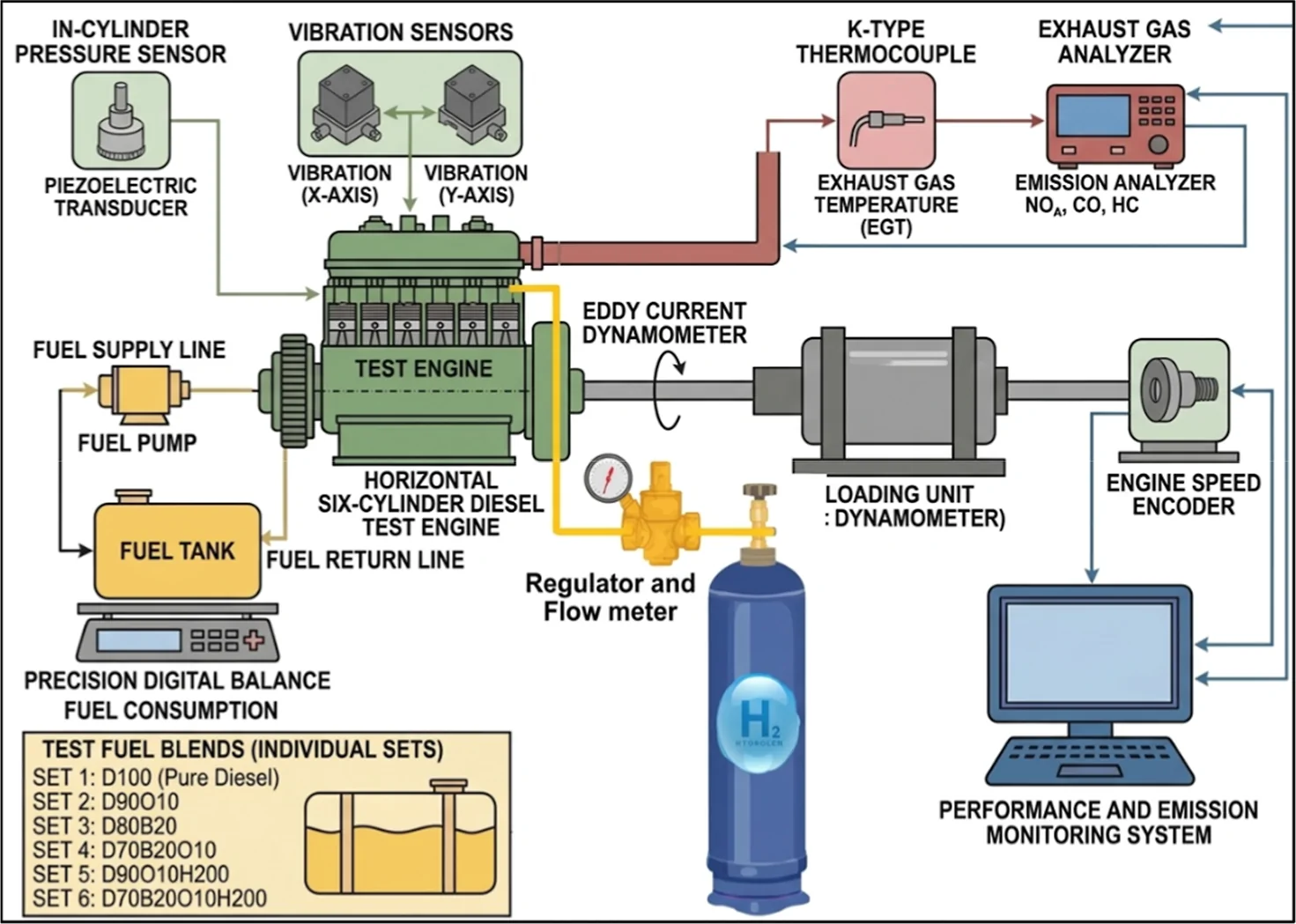
Experimental setup.

A heavy-duty engine with 6 cylinders, 12 L displacement, and a compression ratio of 16.5 was used as the test engine. The technical specifications of the test engine are presented in Table 1.^12^

**Table 1:** Test Engine Properties

displaced volume	12 L
bore	133
stroke	140
cylinders	6 pcs
compression ratio	16.5
max. power	234 HP
max. rpm	2300
max. torque	720 N m
injection timing	16 ′BTDC

Because of the eddy current dynamometer, tests could be performed at different engine loads during the experiments. An eddy current dynamometer with a maximum applicable torque of 600 N m was used in the experiments. A Fenac FN50-360 encoder, producing 360 pulses per revolution, was used to measure the engine speed. A PCB 113B22 piezoelectric pressure sensor was used to collect the in-cylinder pressure data. A K-type temperature sensor was positioned on the engine exhaust manifold for exhaust temperature measurements. A Bosch BAE 60 emission analyzer, whose technical specifications are given in Table 2, was used for exhaust emission measurements.

**Table 2:** Bosch BAE 60 Gas Analyzer

emissions	range	sensitivity
CO	% 0,000–10.00	% 0,001
CO_2_	% 0.00–18.00	% 0.01
HC	0–9999 ppm	1 ppm
O_2_	% 0.00–22.00	% 0.01
NO	0–5000 ppm	1 ppm

A Desis-KF-H2W precision balance with a sensitivity of 0.5 g and a maximum measuring capacity of 5 kg was used to measure liquid fuel consumption. Hydrogen gas was delivered to the combustion chamber from the engine’s intake manifold along with the engine intake air. The hydrogen gas pressure was reduced from 200 to 1 bar using an Alkan 840180 regulator. The gas flow rate was controlled using an Alicat MCR series flow controller.

To measure the engine’s vibration response in the horizontal and vertical axes, two Type 4513B accelerometers were mounted on the engine as shown in Figure 2. Vibration data was collected and recorded using a Brüel & Kjær Type 3560B intelligent data acquisition system.

**Figure 2:**
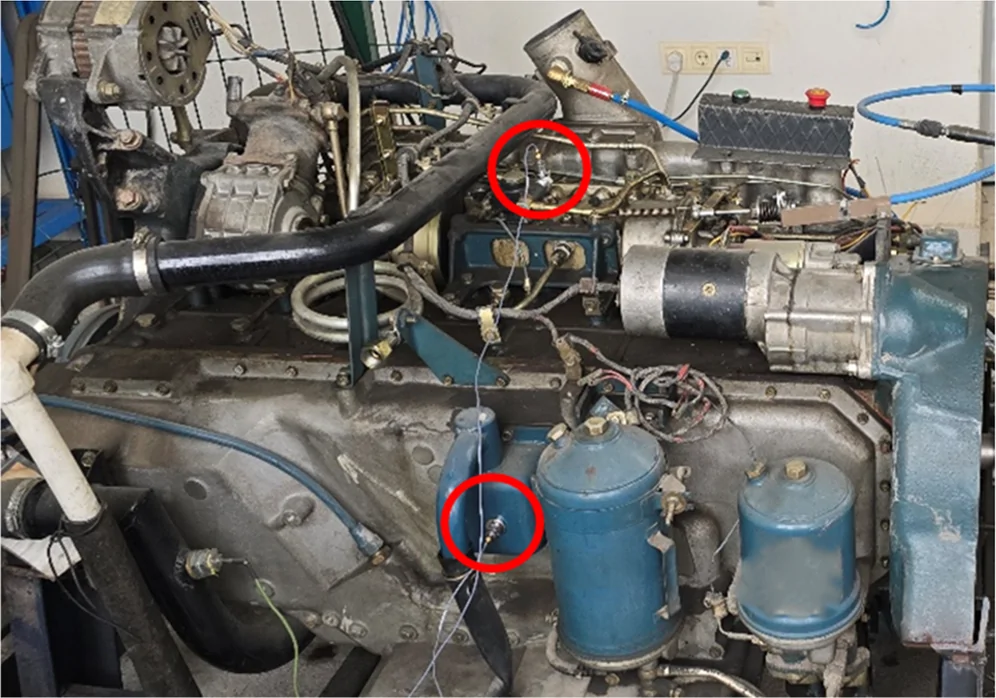
Accelerometers mounted on the motor to measure vibrations in the horizontal and vertical axes.

### 2.2 Fuels

The characteristics of the fuels used in this study are presented in Table 3. The diesel used in the test fuels was commercially available standard diesel (D100). In the experiments, biodiesel obtained from a mixture of 10% spent coffee grounds oil and 90% waste cooking oil (10% produced from SCGO and 90% WCO)^13^ was used. Another liquid fuel additive is octanol. The chemical formula of octanol is C_8_H_17_OH.

**Table 3:** Fuels

specifications	diesel	biodiesel	octanol	hydrogen
density (ρ) at 15 °C, kg/m3	820–845	893.7	825	0.09 (gas)
stoichiometric ratio	14.92	13.8	14.45	34.48 air/fuel
flash point °C	55	130	-	-
autoignition temperature °C	186–230	250–450	270	585
C (%·wt)	86.21	77–78	73.68	0
H (%·wt)	13.79	12	13.91	100
O (%·wt)	0	10–11	12.41	0
viscosity at 40 °C, mm^2^/s	2.5	4.2	7.59	-
cloud point °C	-	–3	-	-
pour point °C	-	0	-	-
cold filter plugging point °C	-	–4	-	-
acid value KOH/g	-	0.45	-	-
free fatty acids %	-	0.225	-	-
iodine value, g iodine/100 g	-	129.56	-	-
cetane number	51	51.16	39.1	-
saponification number, mg KOH/g	-	201.83	-	-
lower heating value, MJ/kg	42.7	37	38.4	120

The preparation of liquid fuel mixtures is shown representatively in Figure 3. Three different liquid fuel mixtures were prepared: D90O10; 90% diesel +10% octanol, D80B20; 80% diesel +20% biodiesel, and D70B20O10; 70% diesel +20% biodiesel +10% octanol. The liquid fuels were mixed with a magnetic stirrer at 500 rpm for 30 min at room temperature. Thus, liquid fuel mixtures were formed. Hydrogen gas, initially at 200 bar pressure, was reduced to a range of 1–1.5 bar with the help of a regulator and then delivered to the combustion chamber from the engine intake manifold at a mass flow rate of 200 g/h (D90O10H200; liquid fuel 90% diesel +10% octanol +200 g/h hydrogen gas. D70B20O10H200; 70% diesel +20% biodiesel +10% octanol +200 g/h hydrogen gas).

**Figure 3:**
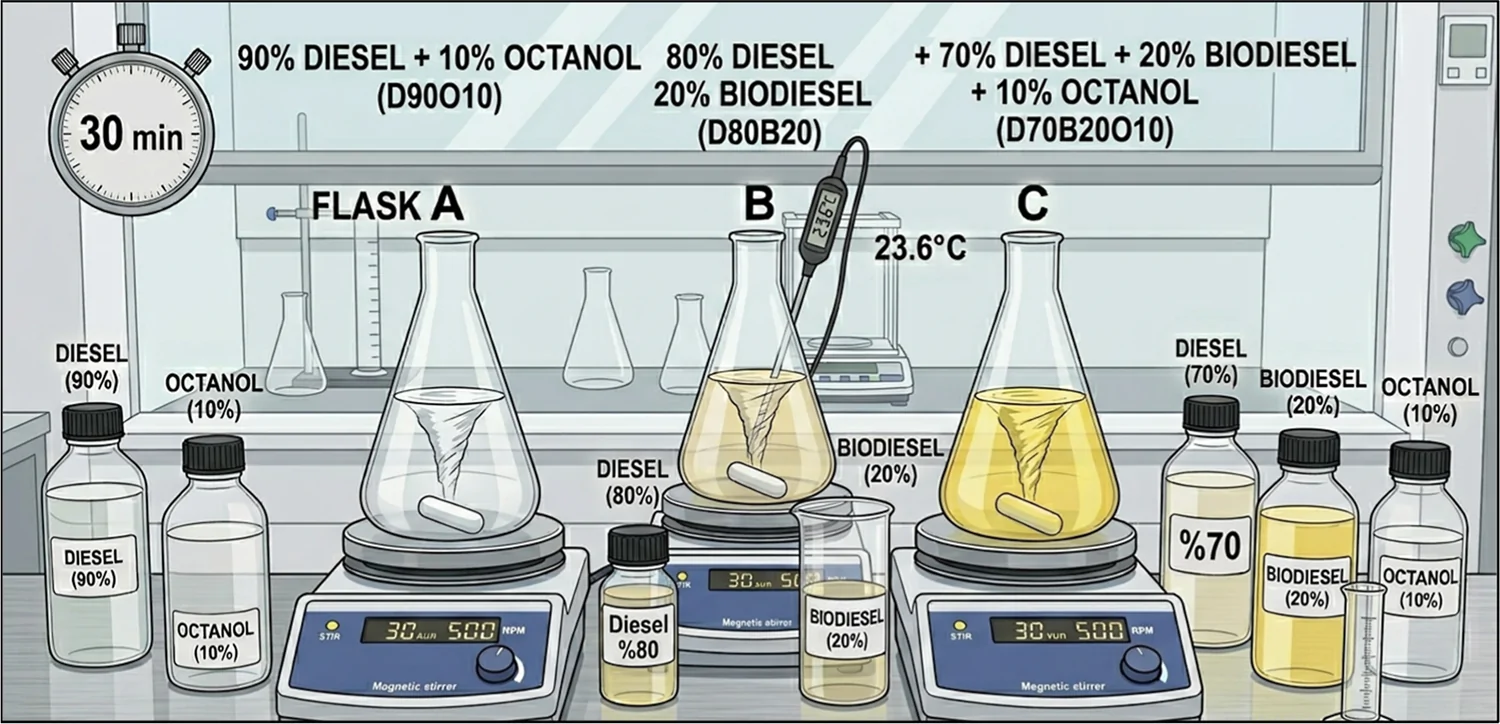
Preparation of test fuels.

### 2.3 Uncertainty Analysis

During the evaluation of engine performance and exhaust emission findings obtained from the experiments, all tests were repeated at least three times and average values were taken into account to avoid errors. To prove the accuracy of the measured parameters, an uncertainty analysis was performed using the least-squares method. The uncertainty ratios of the measurement equipment in the experiments are given. To calculate the total uncertainty, the squares of the percentage values of all equipment in Table 4 were added together and the square root of the sum was taken. With this calculation, the total uncertainty was found to be 2.46%. This value is an acceptable value for the experimental study. The expanded measurement uncertainty was determined as 1.0% in accordance with the EAL-R2 guideline with a coverage factor of *k* = 2 corresponding to a 95% confidence level for a normal distribution.^27–29^

**Table 4:** Uncertainty

instrument	uncertainty
engine BTE measuring system	±1.68%
time	±0.2%
air flow rate	±0.9%
pressure sensor	±1.0%
vibration sensors	±1.0%
CO	±0.2%
HC	±0.4%
NO	±0.4%
total uncertainty	± 2.46%

## 3 Discussion

### 3.1 Engine Performance

#### 3.1.1 BTE and BSEC

BTE is an important performance parameter that measures how much of the fuel energy entering the engine per unit time is converted into useful work. It indicates the engine’s ability to convert thermal energy into mechanical energy. In diesel engines, an increase in the BTE ratio is a significant indicator of increased combustion efficiency and reduced heat losses. BSEC is the total amount of energy the engine requires to produce a unit of power. Comparing BSEC values is the most accurate approach, especially when comparing fuels with different calorific values, as in this study. A low BSEC value indicates that the engine expends less energy to do the same work, i.e., it is more efficient. There is an inverse relationship between BTE and BSEC.^14,15^ In this study, the graph of BTE and BSEC values obtained from the analysis of the experimental data is presented in Figure 4. In addition, the lower calorific values of the test fuel mixtures are presented in Table 5.

**Figure 4:**
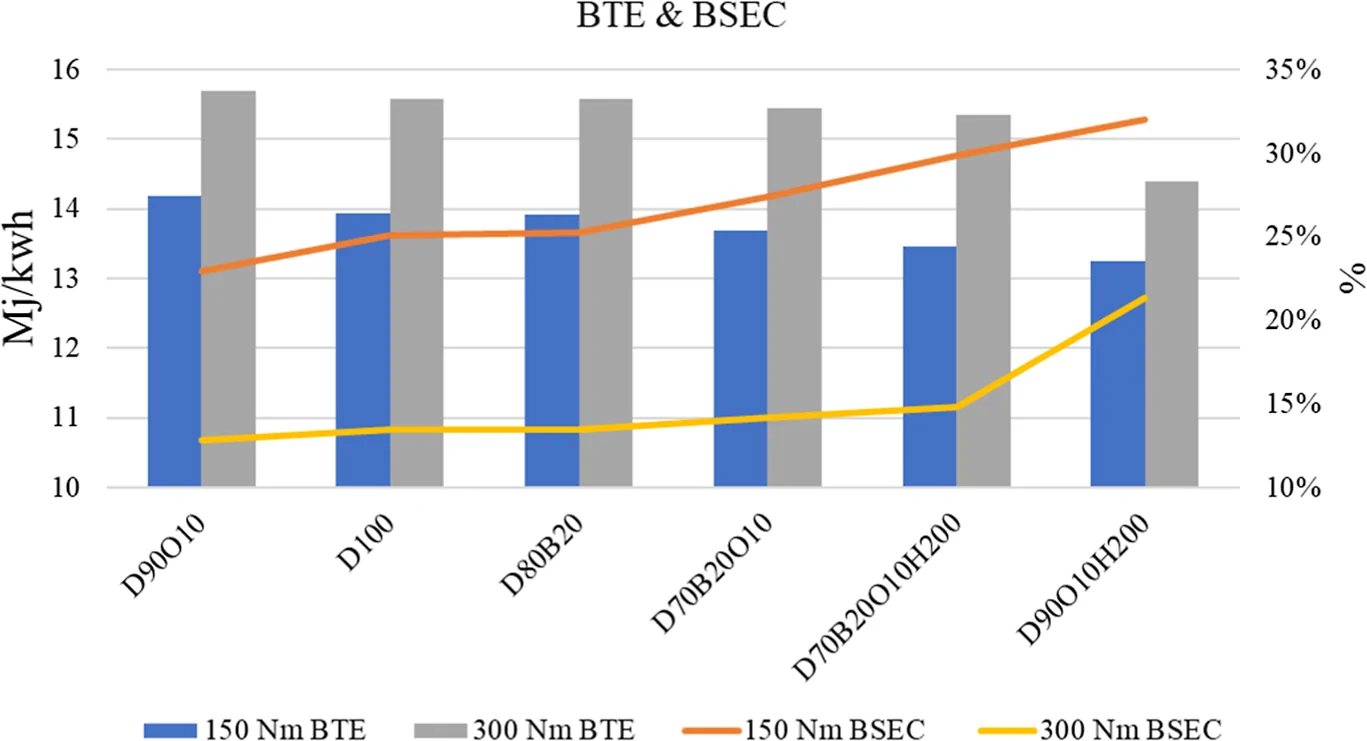
BTE and BSEC.

**Table 5:** LHV Values of Test Fuels

test fuels	LHV MJ/kg
D100	42.7
D90O10	42.27
D90O10H200	46.80
D80B20	41.56
D70B20O10	41.13
D70B20O10H200	45.84

According to the results obtained in this study, a noticeable increase in BTE values was observed in all fuel mixtures when the load increased from 150 N m to 300 N m. The increase in cylinder temperatures and improved combustion efficiency at high loads can explain this. The highest BTE values were obtained in the D90O10 mixture at both low and high loads. The D90O10 test fuel achieved thermal efficiencies of 27.45% at a 150 N m load and 33.72% at a 300 N m load. These values are higher than D100 (BTE; 26.43% at a 150 N m load and 33.24% at a 300 N·m load). This is because the high oxygen content of octanol improves combustion quality. High carbon alcohols such as octanol can improve fuel atomization and ensure complete combustion thanks to their oxygen content. These results are consistent with the study by Ashok et al.^4^ In addition, the observed increase in BTE with the D90O10 blend exhibits a strong consensus with the validation metrics established by ref 5,6 further validating the accuracy of the current experimental setup.

The D80B20 mixture (BTE; 26.35% at 150 N m load and 33.22% at 300 N m load) yielded quite similar results when compared to D100. The slightly lower efficiency of biodiesel-containing mixtures compared with pure diesel can be explained by the higher viscosity of biodiesel and the negative impact on injection quality. Furthermore, the higher combustion resistance of biodiesel compared with that of pure diesel also explains this result. These results are consistent with the studies of Hoang^16^ and Mattarelli et al.^17^ However, a slight decrease in efficiency was observed in the D70B20O10 mixture (BTE; 25.40% at 150 N m load, 32.72% at 300 N m load), where biodiesel and octanol are combined.

When hydrogen was added to the D90O10 fuel, the efficiency decreased from 27.45% to 23.55% at 150 N m and from 33.72% to 28.31% at 300 N m (D90O10H200). This may be because hydrogen, due to its very high combustion rate, causes the end-of-combustion pressures to peak well before top dead center, thus generating negative work and reducing thermal efficiency. It is also thought that the decrease in the thermal efficiency may be due to the decrease in the volumetric efficiency. Saravanan and Nagarajan^18^ stated that in studies where hydrogen was injected into the intake manifold, the efficiency decreased at low loads, but at high loads, hydrogen could increase the efficiency by accelerating diffusion combustion. In this study, it is also considered that the hydrogen additive creates a very early combustion phase in the combustion chamber. Therefore, it is thought that the thermal efficiency of the hydrogen-added tests was low.

#### 3.1.2 In-Cylinder Pressure

In-cylinder pressure and net heat release are important indicators for understanding the combustion quality in internal combustion engines. In diesel engines, the starting point of fuel injection, fuel quantity, amount of air in the cylinder, ignition delay time, chemical properties of the fuel, compression ratio, temperature, and pressure in the cylinder are factors that directly affect the cylinder top pressure. Therefore, measuring in-cylinder pressure in experiments provides important information about the combustion characteristics of the fuel in the cylinder.^23^

In this study, the in-cylinder pressure graphs obtained for 150 N m and 300 N m are shown in Figures 5 and 6. According to the graph, the ignition of the test fuels containing biodiesel was delayed compared to standard diesel at both 150 N m and 300 N m loads. In addition, the combustion pressures of fuels containing biodiesel are lower than those of pure diesel. One of the reasons for this is that the viscosity of biodiesel is higher than that of standard diesel. This situation reduces the atomization ability of the fuel [57]. Another reason is that biodiesel draws more heat from the cylinder to vaporize compared to pure diesel. Therefore, the cylinder interior is relatively cooler than pure diesel fuel, causing a drop in cylinder pressure. Consequently, the thermal efficiency of test fuels containing biodiesel is also lower than that of pure diesel.^24–26^ D9010 fuel exhibited an optimum pressure graph, particularly at a load of 300 N m. This explains why D90O10 fuel had the highest thermal efficiency. However, hydrogen supplementation caused the combustion peak pressure to shift significantly before top dead center, producing negative work and negatively impacting thermal efficiency.

**Figure 5:**
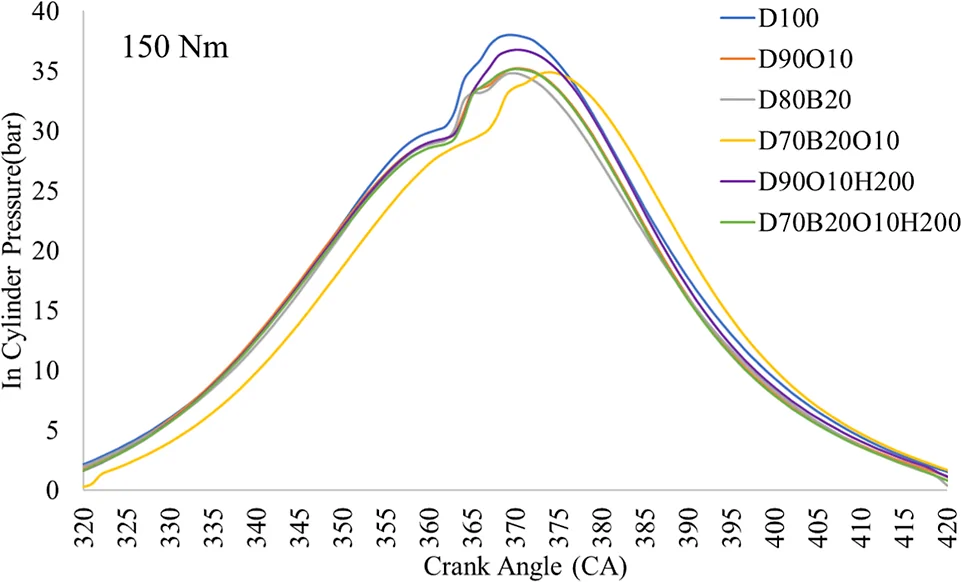
In-Cylinder pressure graph at 150 N m load.

**Figure 6:**
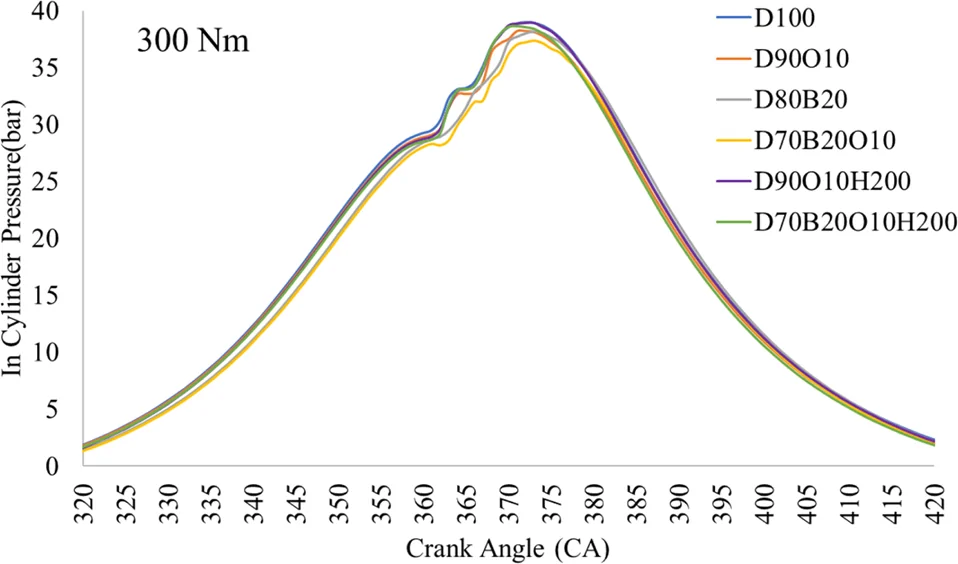
In-Cylinder pressure graph at 300 N m load.

In summary, while 10% octanol additive to pure diesel fuel positively impacted engine performance, biodiesel and hydrogen additives negatively affected the thermal efficiency for the reasons described above.

### 3.2 Emissions

#### 3.2.1 Carbon Monoxide

Looking at Figure 7, the most striking finding is the significant reduction in CO emissions across all fuel types, depending on the increase in engine load. This is because, at high loads, cylinder temperatures and pressures increase. Thanks to the increased temperature and pressure, air and fuel burn more homogeneously. This accelerates the oxidation of CO to CO_2_, reducing incomplete combustion products.^12,19,20^

**Figure 7:**
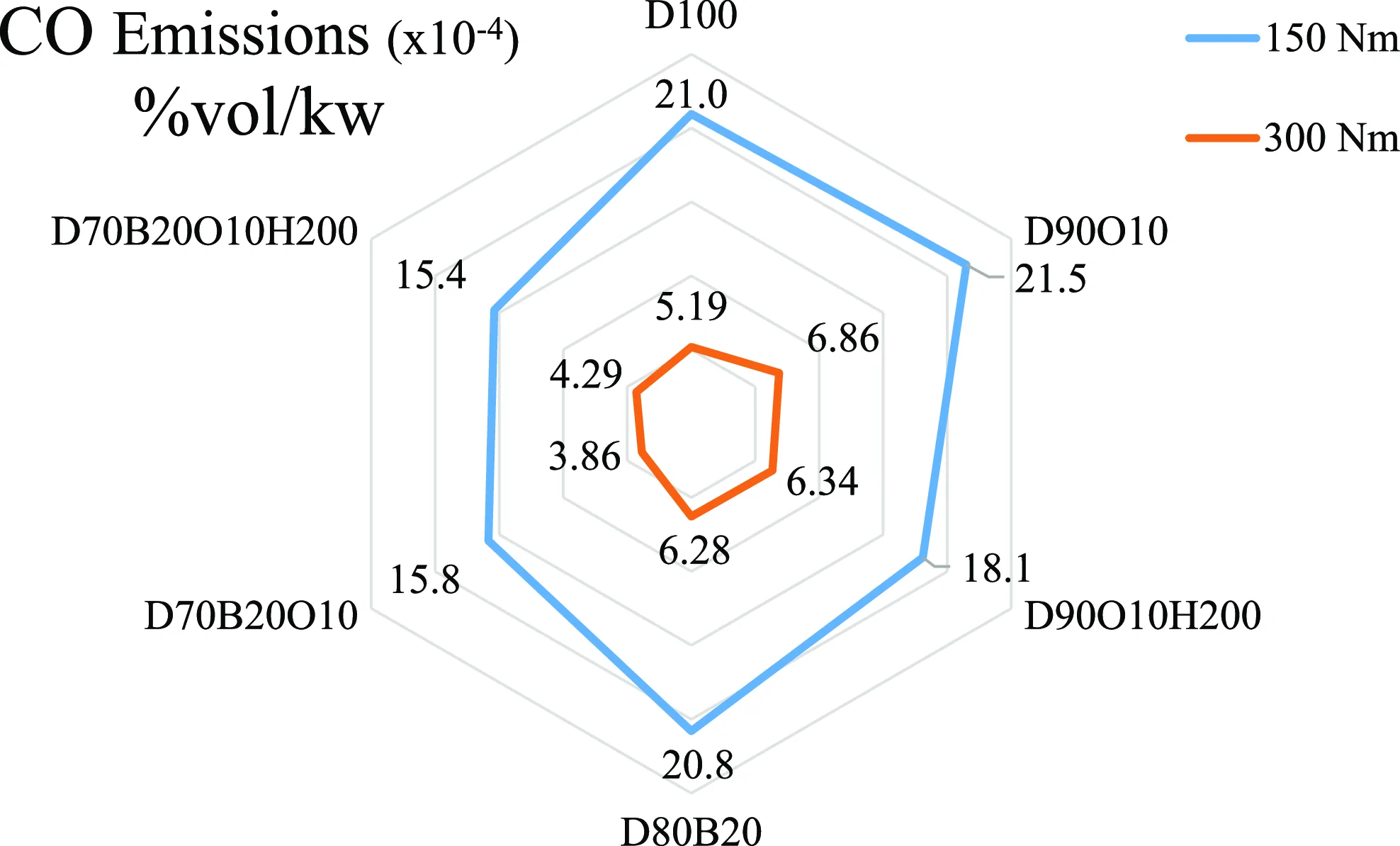
CO emissions (%vol/kw).

The D70B20O10 mixture is seen to produce lower CO emissions than D100 under both load conditions. The high oxygen content in biodiesel and octanol minimizes the oxygen-depleted parts in the combustion chamber by supplying oxygen from within the fuel itself. This helps to oxidize a greater amount of carbon monoxide and convert it to carbon dioxide. This improves the CO emissions. Furthermore, alternative multifuel formulations, particularly those incorporating biodiesel matrices, significantly dictate complex exhaust gas properties and nanoparticle behavior in varying thermal and chemical environments, as recently mapped by ref 34.

Since hydrogen is a carbon-free fuel, it does not directly contribute to CO formation. Furthermore, hydrogen’s high flame rate and wide ignition limits improve the combustion quality of diesel/biodiesel blends, the main fuel in the cylinder, aiding in CO oxidation through a chain reaction. Therefore, hydrogen plays a significant role in reducing CO emissions. In particular, the D70B20O10H200 blend has yielded one of the lowest CO values at low load.

#### 3.2.2 Nitrogen Oxide

As shown in Figure 8, NO emissions increase as the load increases. This is because an increase in load means that higher temperatures are generated inside the cylinder.^21,22^ Since NO formation is extremely sensitive to temperature, the increase in temperature at high loads has increased NO emissions. It is observed that the D80B20 and D70B20O10 mixtures produce more NO than D100 at both load levels. This is because oxygen in the structure of biodiesel and octanol creates oxygen-rich regions during combustion. This triggers the oxidation of nitrogen and accelerates the NO formation.

**Figure 8:**
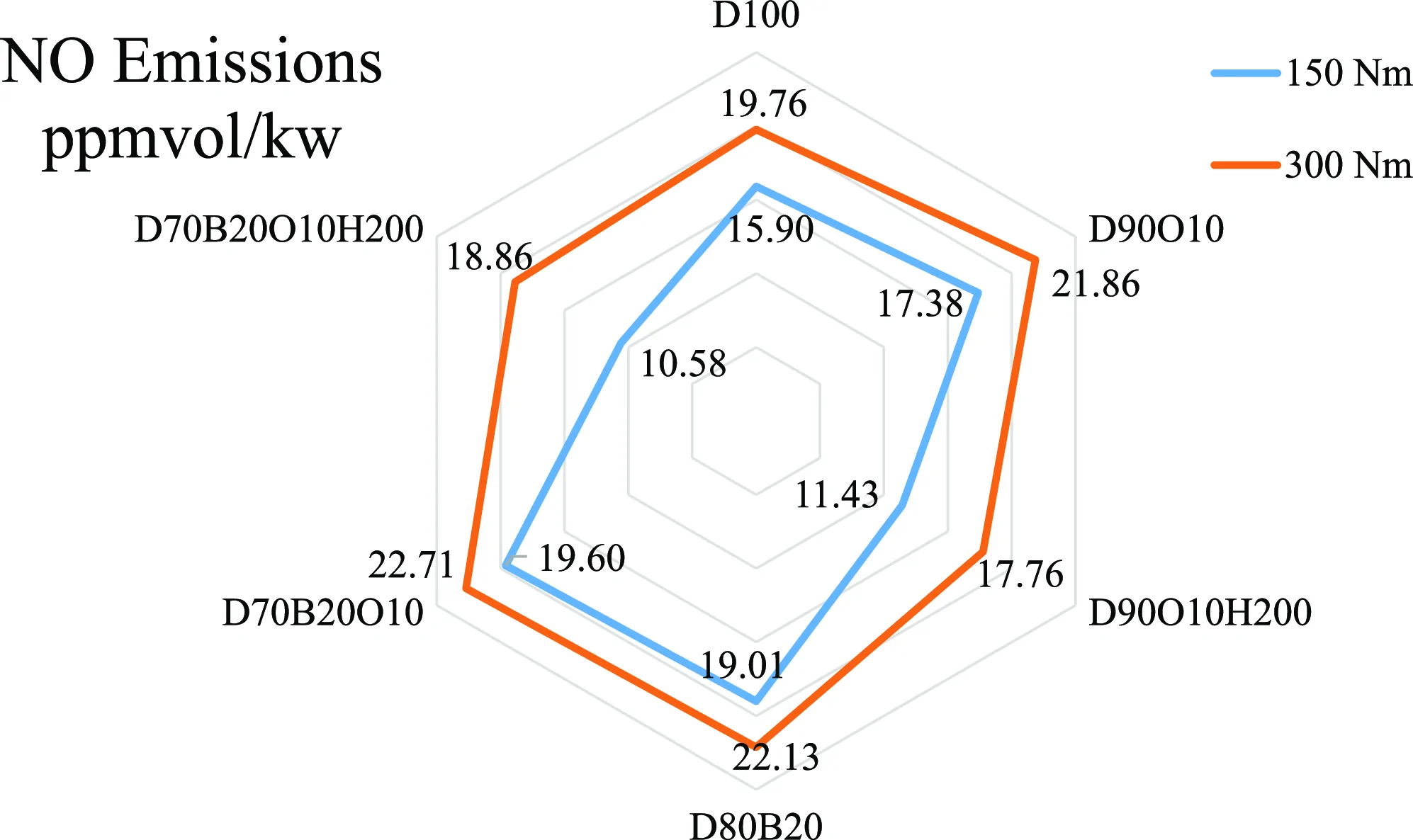
NO emissions (ppmvol/kw).

The addition of hydrogen significantly reduced the level of NO emissions. Comparing D70B20O10 (19.60 ppm/kW) to D70B20O10H200 (10.58 ppm/kW), there was approximately a 46% reduction. This can be explained in two ways: First, hydrogen replaces a portion of the intake air when fed through the intake manifold. This reduces the total amount of oxygen in the combustion chamber and somewhat suppresses the combustion temperature. Second, the presence of hydrogen may have altered the ignition delay and combustion characteristics of the base fuel, creating “cold combustion” zones or shifting peak pressure points. It is thought that the hydrogen sent from the intake manifold to the combustion chamber reduces NO by sweeping away a certain amount of intake air and thus lowers the oxygen level.

#### 3.2.3 Hydrocarbon (HC) Emissions

In all fuel types (Figure 9), HC emissions are observed to decrease dramatically when the engine load increases from 150 N m to 300 N m. An increase in in-cylinder temperatures at high loads increases the combustion rate, ensuring complete combustion of the fuel. In addition, the flame extinction distance in the regions near the cylinder walls shortens with the increase in temperature, which reduces the amount of unburned fuel.^15,20^

**Figure 9:**
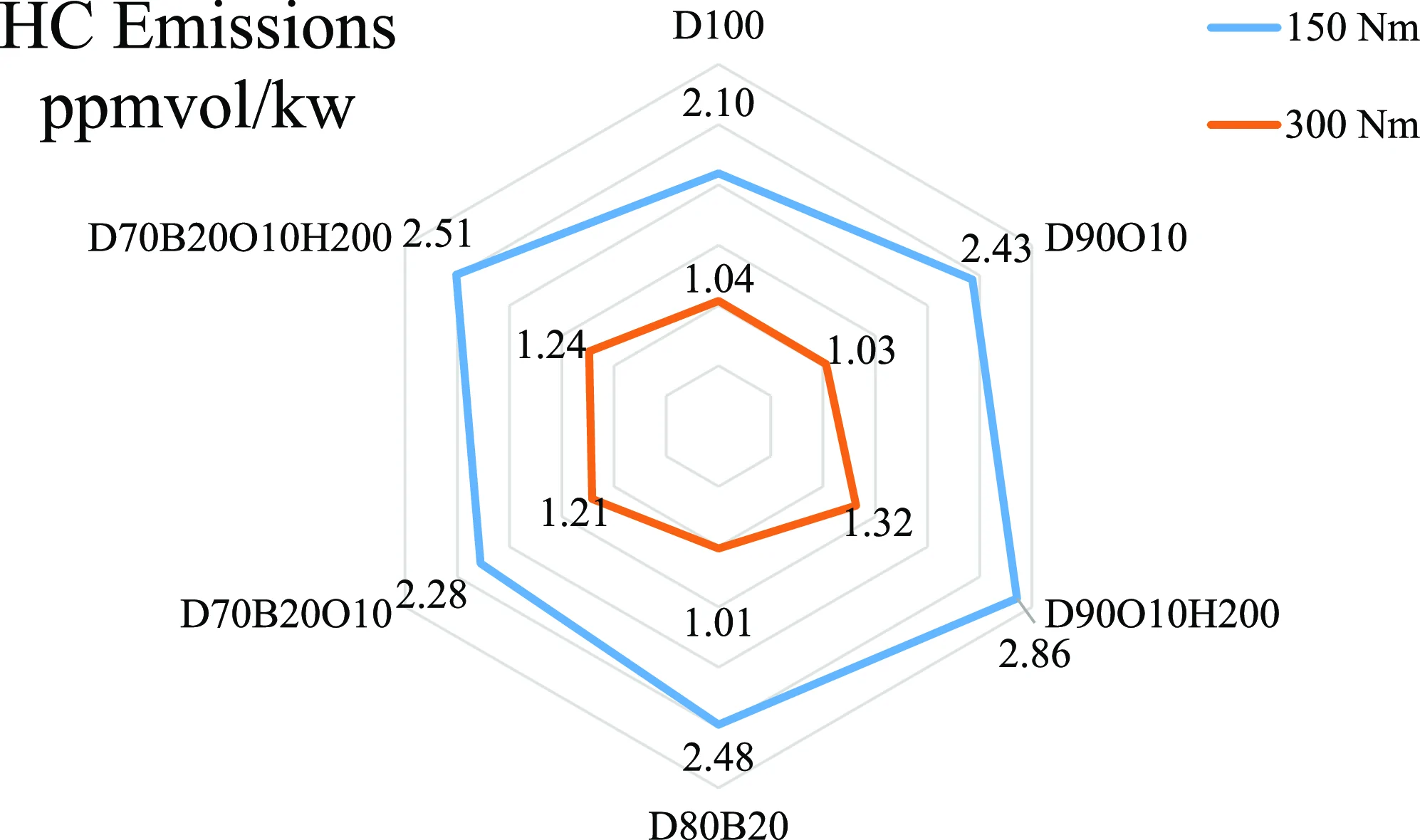
HC, Hydrocarbon (ppmvol/kw) emissions.

Blends such as D90O10 and D80B20 produced higher HC emissions than pure diesel at 150 N m load. This is due to the high latent heat of vaporization of alcohols. At low loads, this can cool the in-cylinder temperature somewhat, leading to HC formation. Another reason is that the cetane number of alcohols is lower than that of diesel, which can prolong the ignition delay, causing the fuel to reach the combustion chamber walls and extinguish the flame. However, at 300 N m load, oxygen content becomes dominant, reducing HC values to diesel levels. The addition of hydrogen increased HC emissions somewhat. Although hydrogen does not contain carbon, when introduced through the manifold in diesel engines, it replaces the intake air. This can make it more difficult for fuels to burn completely in the cylinders. Therefore, it may have caused an increase in HC emissions.

### 3.3 Vibration

In this study, the test engine was a diesel engine with six horizontally positioned cylinders. This information is considered crucial for the reader when analyzing the vibration data. This is because the variation in horizontal vibrations in this study is primarily related to the up-and-down movement of the piston and the forces acting in the piston direction, resulting from combustion. Because of the horizontally positioned cylinders of the engine, most of the vertical vibrations may be caused by the lateral forces applied to the cylinders during ignition and the imbalance of the crankshaft. In summary, the direction of vibration affected by the combustion characteristics of the test fuels in this study is the horizontal direction, because the test engine is a diesel engine with horizontally positioned cylinders. The vibration data obtained in this study are presented in Tables 6 and 7. Vibration graphs are also shown in Figures 10 and 11.

**Table 6:** Engine Vibration and Root Mean Square (RMS) Values for 150 N·m

test fuels	RMS value (horizontal)	RMS value (vertical)	resultant vibration
D90O10H200	41.5376	37.8368	56.187
D100	32.9034	40.0869	51.861
D80B20	28.8428	38.8904	48.419
D70B20O10H200	31.3511	36.7963	48.341
D70B20O10	27.9093	37.0086	46.353
D90O10	26.5049	37.8142	46.178

**Table 7:** Engine Vibration and RMS Values for 300 N·m

test fuels	RMS value (horizontal)	RMS value (vertical)	resultant vibration
D70B20O10H200	47.0129	46.9623	66.450
D80B20	37.6158	44.3863	58.181
D70B20O10	37.7423	44.1926	58.116
D90O10H200	33.9760	41.5376	53.663
D90O10	33.6497	42.3644	54.102
D100	31.6339	41.0318	51.810

**Figure 10:**
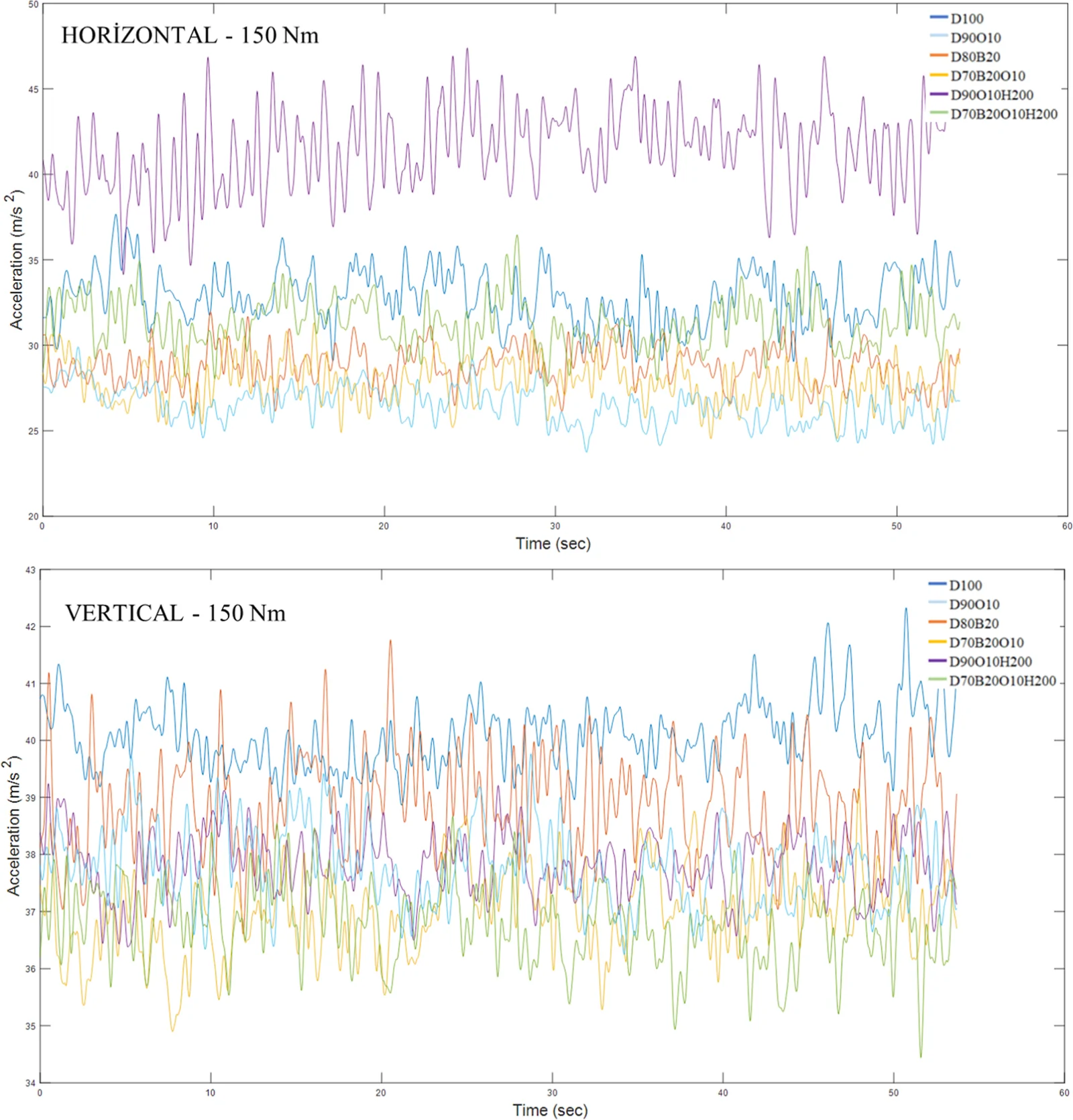
Vibration graph at a load of 150 N·m.

**Figure 11:**
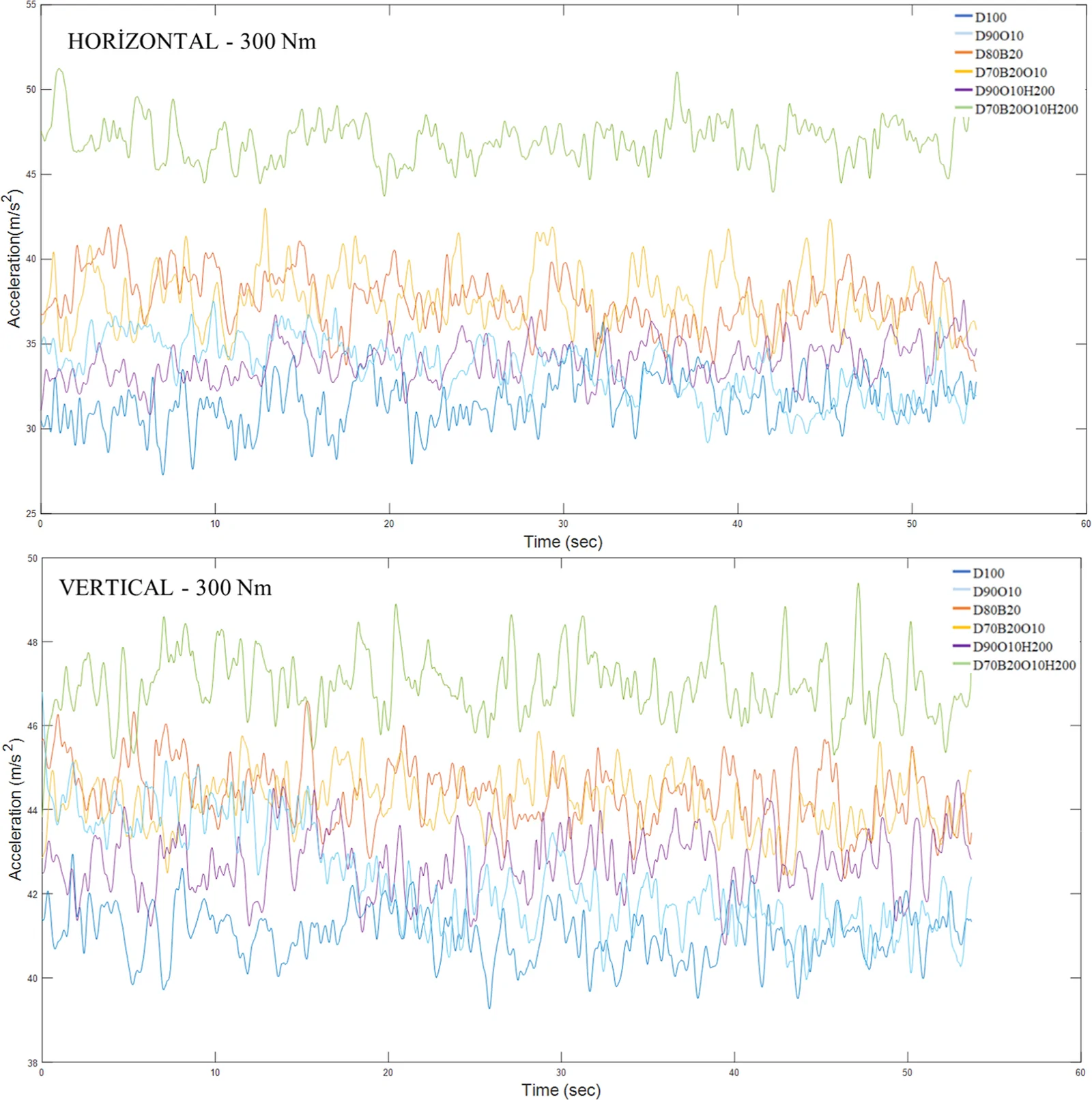
Vibration graph at a load of 300 N·m.

To evaluate the destructive power of the measured vibration forces and the heat energy they generate, the Root Mean Square (RMS) calculation was used. RMS is a vital parameter representing the total energy content of a vibration signal. Mathematically, RMS is the square root of the average of the squares of all instantaneous values in the vibration waveform. The formula is shown in eq 1. Here, *f*(*t*) represents the amplitude of the vibration at that moment as a function of time. T represents the total time interval during which the measurement is taken, or the period in which the signal repeats itself. The smallest unit of time is represented by dt (differential time).

1 $$RMS=\sqrt{\frac{1}{T}\int_{0}^{T}{\left[f\left(t\right)\right]}^{2}dt}$$

The resultant vibration value was calculated as shown in eq 2.

2 $$resultant\;vibration=\sqrt{x_{RMS}^{2}+y_{RMS}^{2}}$$

When the engine load increased from 150 N m to 300 N m, the vibration intensity increased in almost all fuel types. As the load increases, the increase in the cylinder pressure and combustion intensity naturally leads to increased mechanical vibration on the engine block. This increased engine block vibration directly increased the RMS values. For example, in the D70B20O10H200 mixture, the resultant vibration was 48,341 at 150 N·m, while it increased to 66,450 at 300 N·m.

Hydrogen has a very high combustion rate. When the experimental results are evaluated, it is seen that the addition of hydrogen has a vibration-enhancing effect, especially at high loadings. The rapid flame propagation rate of hydrogen increased the rate of increase in the cylinder pressure (especially at 300 N m load). This created a “knocklike” effect, raising the vibration levels. The highest resultant vibration value at 300 N m load was observed in the D70B20O10H200 fuel containing hydrogen.

Biodiesel and octanol blends have generally been observed to improve (in terms of reducing vibration) vibration at low loads (150 N m) compared to pure diesel. However, at 300 N m, biodiesel and octanol blends also showed a vibration-enhancing effect compared to pure diesel. While the combined vibration of D100 at 150 N m was 51.861, blends containing octanol and biodiesel (D90O10, D70B20O10) were found to be below this value (to levels of 46.178 and 46.353, respectively). At low loads, the high cetane number of biodiesel and the moderating effect of octanol on combustion can dampen the mechanical vibration by providing a smoother combustion process.

In summary, adding hydrogen to the fuel mixture creates a disadvantage in terms of engine mechanical vibration, especially at high loads. Pure diesel and simple octanol blends exhibited a more stable operation at high loads.

## 4 Conclusion

In this study, test fuels were created using combinations of diesel, biodiesel (%10 produced from SCGO and %90 WCO), octanol, and hydrogen. Hydrogen was delivered to the combustion chamber via the intake manifold at a mass flow rate of 200 g/h. Furthermore, the effects of the test fuels on engine vibration were investigated using accelerometers placed on the horizontal and vertical axes of the engine. The results obtained from this study are summarized below.

1 The highest BTE values were obtained in the D90O10 mixture at both low and high loads. The D90O10 test fuel achieved thermal efficiencies of 27.45% at a 150 N·m load and 33.72% at a 300 N·m load. These values are higher than D100 (BTE; 26.43% at a 150 N·m load and 33.24% at a 300 N·m load).
2 The D80B20 mixture (BTE; 26.35% at 150 N·m load and 33.22% at 300 N·m load) yielded quite similar results when compared to D100. However, a slight decrease in efficiency was observed in the D70B20O10 mixture (BTE; 25.40% at 150 N·m load, 32.72% at 300 N·m load), where biodiesel and octanol are combined.
3 When hydrogen was added to the D90O10 fuel, the efficiency decreased from 27.45% to 23.55% at 150 N·m and from 33.72% to 28.31% at 300 N·m (D90O10H200). This may be because hydrogen, due to its very high combustion rate, causes the end-of-combustion pressures to peak well before top dead center, thus generating negative work and reducing thermal efficiency.
4 The D70B20O10 mixture is seen to produce lower CO emissions than D100 under both load conditions. The high oxygen content in biodiesel and octanol minimizes the oxygen-depleted parts in the combustion chamber by supplying oxygen from within the fuel itself. This helps to oxidize a greater amount of carbon monoxide and convert it to carbon dioxide. This improves the CO emissions.
5 It is observed that the D80B20 and D70B20O10 mixtures produce more NO than D100 at both load levels. This is because oxygen in the structure of biodiesel and octanol creates oxygen-rich regions during combustion. This triggers the oxidation of nitrogen and accelerates the NO formation.
6 The addition of hydrogen significantly reduced the level of NO emissions. Comparing D70B20O10 (19.60 ppm/kW) to D70B20O10H200 (10.58 ppm/kW), there was approximately a 46% reduction. This can be explained in two ways: First, hydrogen replaces a portion of the intake air when fed through the intake manifold. This reduces the total amount of oxygen in the combustion chamber and somewhat suppresses the combustion temperature. Second, the presence of hydrogen may have altered the ignition delay and combustion characteristics of the base fuel, creating “cold combustion” zones or shifting peak pressure points.
7 Blends such as D90O10 and D80B20 produced higher HC emissions than pure diesel at a 150 N m load. This is due to the high latent heat of vaporization of the alcohols. At low loads, this can cool the in-cylinder temperature somewhat, leading to the formation of HC. The addition of hydrogen increased the HC emissions somewhat. Although hydrogen does not contain carbon, when introduced through the manifold in diesel engines, it replaces the intake air. This can make it more difficult for the fuels to burn completely in the cylinders.
8 When the engine load increased from 150 N m to 300 N m, the vibration intensity increased in almost all fuel types. For example, in the D70B20O10H200 mixture, the resultant vibration was 48,341 at 150 N m, while it increased to 66,450 at 300 N·m.
9 Hydrogen has a very high combustion rate. When the experimental results are evaluated, it is seen that the addition of hydrogen has a vibration-enhancing effect, especially at high loadings. The highest resultant vibration value at 300 N·m load was observed in the D70B20O10H200 fuel containing hydrogen.
10 Biodiesel and octanol blends have generally been observed to improve (in terms of reducing vibration) vibration at low loads (150 N m) compared to pure diesel. However, at 300 N·m, biodiesel and octanol blends also showed a vibration-enhancing effect compared to pure diesel. While the combined vibration of the D100 at 150 N·m was 51.861 mm/s^2^, blends containing octanol and biodiesel (D90O10 and D70B20O10) were found to be below this value (to levels of 46.178 mm/s^2^ and 46.353 mm/s^2^, respectively).

The most important findings from this study are that the addition of 10% octanol to pure diesel fuel improves thermal efficiency. Significant findings regarding vibration include the fact that biodiesel-containing blended fuels reduce vibration compared with pure diesel fuel at low loads. However, at high loads, the vibration increased slightly in biodiesel-containing fuels. Hydrogen supplementation showed an increasing effect on vibration at both 150 N m and 300 N m loads.

## 5 Future Perspectives

The physical implementation of the four fuel blends examined in this study offers a viable way for standard compression ignition engines to utilize high-rate alternative fuels without requiring major structural changes. The dual benefit of achieving a higher brake thermal efficiency (BTE) with an octanol blend and simultaneously using hydrogen and octanol supplementation to reduce engine vibrations, especially at high loads, provides a viable framework for developing cleaner, structurally stable, and efficient dual-fuel propulsion systems.

These findings demonstrate that octanol can act as an effective stabilization agent for biodiesel–diesel matrices. At the same time, targeted hydrogen injection can offset the small increases in structural vibration typically observed under high load conditions.

For future research, several potential avenues can be explored to advance the discourse. Long-term durability and wear analysis is one such avenue. Another is advanced CFD and combustion modeling. The use of advanced computational fluid dynamics (CFD) for precise mapping is a significant research method. Finally, the goal is to advance research in dynamic vibration control.

## Nomenclature

BSEC: brake specific energy consumption
BTE: brake thermal efficiency
N·m: Newton meter
RMS: root mean square
SCGO: spent coffee grounds oil
WCO: Waste cooking oil
ppm: parts per million
CO: carbon monoxide
HC: hydro carbon
NO: nitrogen oxide
LHV: lower heating value
rpm: revolution per minute
H_2_: hydrogen
